# Recognition of Children’s Learning in Educational Research, Policy and Practice: Herbison Invited Lecture, NZARE Annual Conference 2022

**DOI:** 10.1007/s40841-023-00276-5

**Published:** 2023-01-30

**Authors:** John O’Neill

**Affiliations:** Massey University, Palmerston North, New Zealand

**Keywords:** educational research, children learning, recognition, reification, alienation

## Abstract

For Jean Herbison, learning in her early 20th century childhood world was relatively uncomplicated and predictable. Life was shaped by unambiguous family, faith and settler colonial prescriptions about how children *should* behave and what they should become. Approaching the centenary of her birth, children today must navigate a very different society of ‘unlimited *can’*; an achievement society that generates a debilitating compulsion to self-improve (Byung Chul-Han).

In this Herbison lecture, I offer a personal reflection on the contemporary ‘triangle’ of education research, policy and practice in Aotearoa New Zealand. Viewed as a culturally and historically specific ‘form of life’ (Rahel Jaeggi), I ask whether, over the last thirty five years, this triangle may have unwittingly contributed to a collective failure to give adequate recognition to children’s learning. Despite our best intentions, have we simply reified students and in doing so alienated them from learning in all its complexities and dimensions (Knud Illeris)?

More than mere acknowledgement of ‘the other’, recognition theory highlights the importance of socially developed qualities such as confidence, respect and esteem (Axel Honneth) to each child’s capacity to develop meaningful relationships to or ‘resonance’ with an ever accelerating and uncontrollable world (Hartmut Rosa) and the people and communities in it. In practical terms, then, what can we draw on that is already immanent in our research, policy and practice triangle to transform children’s institutionalised learning?

## Introduction

E ngā mana, e ngā reo, e ngā maunga, e ngā awaawa, e ngā pataka o ngā taonga tuku iho. Tēnā koutou katoa.

I am very grateful to the Council for this opportunity to honour Dame Jean Herbison’s contribution to education. Much of what I shall say today has been shaped by research collaborations and casual conversations about schooling with critically minded colleagues over the years. I’d like to acknowledge three in particular: Ivan Snook for his courageous defence of teaching as an ethical and relational practice; Roseanna Bourke for her infectious curiosity about children’s learning and assessment in the round; and, much more recently, he wāhine toa, Mere Berryman, for her hopeful endurance against the iron cage of Pākehā education bureaucracy. Whether I stand on their shoulders or in their shadows, I leave for you to decide.

I never met Jean Herbison: she was ‘before my time’, as it were, and she left no lasting research footprint, as such. I know little about her decades as a senior leader and manager in teacher education, vocational training, university governance or national education planning — breaking through masculinist glass ceilings, as she did, with each career advancement. In thinking about how to honour her motivations and achievements, I was fortunate to find a two-and-a-half-hour oral history recording from the Alexander Turnbull Library’s Dames Oral History Project.^1^

As such treasures often do, her ‘remembrances’ as she called them brought Jean Herbison’s early living and learning into sharp relief as she reflected in 1993 at the age of 70, on how she became who she was and what she achieved after leaving school, very reluctantly at age 15, to become a junior office administrator. I now know, for instance, that as a secondary schoolteacher and, later, guidance counsellor, in Christchurch, Jean Herbison was drawn to the ‘non-academic’ adolescents and that, somewhat against the grain of the time (the 1950s), she introduced developmentally oriented, collaborative, project-based curricula to enable her students to meaningfully link schooling, everyday life, local community and the world of work.

I suspect, then, that Dame Jean would be greatly interested in how contemporary education research, policy and practice collectively position children and childhood and more particularly, the ways in which schooling practically assists, or not, with equipping them for their psychological, social and economic worlds. In that spirit, this address will consider:

(i) the complexities of today’s childhood worlds;
(ii) the (im)possibilities of research, policy and practice as triangle; and an alternative conception of this cluster of related activities as a ‘form of life’. It will then
(iii) draw on recognition theory as a vital counter to our unfortunate reification and alienation of children from their schooling; and, finally, attempt to
(iv) ‘walk backwards into the future’ of schooling to identify education research, policy and practice insights from our past and present that might afford us greater recognition of children’s learning in the future.

In this first part, I want to draw a comparison between the comparatively unadorned, predictable, and ‘low-tech’ world of young Jean Herbison in the 1920 and 1930s, and the more complex, multifaceted lives of many children growing up today.

### Learning

In the broadest sense of the term, living is itself a learning experience.

*Humans are created as learners*. But we are at the same time also *doomed to be learners*, we have no possibility to avoid learning, although we do not always learn exactly what we ourselves or others have intended. In contemporary societies, we are also *enforced to be learners*. In nearly all countries there is compulsory school attendance, and in addition to this there is a lot that we all have to acquire in order to be able to function in daily life and various specific contexts. It is to a great extent this direct as well as indirect enforcement that in various connections can make learning problematic. We cannot restrict ourselves to learn what we like or meet by chance. Learning is both an individual and a societal matter. (Illeris, 2017, p. 1)

By looking back from our 21st-century standpoint to Jean Herbison’s childhood learning in early 20th century, British Dominion Dunedin and Invercargill, we may be able more easily to ‘make the familiar strange’ as C. Wright Mills (1959) put it and adopt a reflexive stance toward the often taken-for-granted ways in which children today navigate the demands of both direct and indirect enforcement to learn.

In this regard, the Korean-born German philosopher Byung-Chul Han (2015) draws a useful distinction between ‘the obedience-subject’ and the ‘achievement-subject’ in his essay, *The Burnout Society*.

Today’s society is no longer Foucault’s disciplinary world of hospitals, madhouses, prisons, barracks, and factories. It has long been replaced by another regime, namely a society of fitness studios, office towers, banks, airports, shopping malls and genetic laboratories. Twenty-first-century society is no longer a disciplinary society but an achievement society. Also, its inhabitants are no longer “obedience-subjects” but “achievement-subjects.” They are entrepreneurs of themselves. (p. 8)

For Han, disciplinary society was negative, characterised by authority, rules, constant ‘may not’ prohibitions, and compulsive ‘should’ exhortations (pp. 8 & 9). In these terms, Jean Herbison’s child and adolescent upbringing was most certainly that of an obedience-subject.

## Disciplinary Society and Childhood

In her interview, Herbison recounted the personal philosophy that had sustained her through a long and varied career as a commercial office administrator, school principal’s secretary, teacher, counsellor, teacher educator and senior tertiary education executive and governor:

I’ve always had a philosophy of why I was doing things. I guess it is to know yourself, accept yourself and be yourself. I now know that for me the potential to be my best self is in here. Now I don’t always achieve it but it’s there and I just try as hard as I can, failing as I do in many ways to carry that out.

So, what can we glean from the structural and cultural backcloth to Jean’s everyday learning in her childhood world that may have helped form her approach to life and work in her adult world?

First, perhaps, that her childhood world was essentially mono-ethnic. Two years before she was born, in the *Appendices* to the 1921 census, out of a total recorded Māori population of 52,751 there were only 2,088 persons in the South Island as a whole and approximately 80 in the Counties that make up today’s greater Dunedin area (Census and Statistics Office, 1921). In 1938, the year Jean left school, *Littledene*, a much-lauded sociological study of rural life in Canterbury published four years after the establishment of the New Zealand Council for Educational Research, made only one casual mention of Māori, to the effect that before ‘the white man’ cleared and settled the area, ‘the Maoris’ made frequent hunting raids but never permanently settled (Somerset, 1974, p. 6). Not surprisingly, then, until she took up a senior role at the technical institute in Christchurch in the 1970s (her 50s), the only contact Jean could recall with Māori was in the early 1930s. Four orphaned grandchildren of a Cook Island Māori family came to live for a while next door before they were sent to the orphanage when their grandmother could no longer cope (The children were Alastair [Ariki] Campbell, his younger brother and elder sisters).

Second, her childhood world was a product of the interwar and Great Depression years before the public health and welfare protections introduced through the Social Security Act of 1938. Jean described a close-knit, normal family that enjoyed each other’s company, despite their limited means. Jean’s policeman father (b. 1884) bought a gramophone and car that provided the family with broader cultural and geographical experiences. Yet one of her brothers also remembered him taking three 10% wage cuts during the Depression. Neither parent went beyond primary schooling but each of the children was allowed to attend secondary school “up to a point”. In Jean’s case, the family had moved to Invercargill when she was 12 and she had to move from the (more academic) languages stream at Otago Girls’ High School to the commercial stream at Southland Girls. At the insistence of her father, she reluctantly left secondary school in 1938 to take up an office secretarial position he had found for her in town.

And I just wept my heart out for days, day after day, about that and used to go back to the school and stand around at lunchtimes and that kind of thing, just pining for going back again.

Third, hers was a patriarchal, disciplinarian household environment with a strictly gendered division of both labour and leisure. Jean’s mother (b. 1889) gave birth to six children between 1918 and 1926 and was “worn out really by having children” to the point where in the 1930s she had a “kind of breakdown” at home that required a neighbour to come in and sit with her, and later several other illnesses when one or other of the children had to come home from school to do the same. Jean could recall the gendered functionality of the family home at 126 Richardson Street, St Clair, in particular the two main ‘homemaking’ domains. One was the kitchen and laundry (her mother’s):

During the time when the family all grew up mother had a washerwoman who came every Monday and did the washing for her, but I think that was about all. She might have helped in the house, but mother would certainly have needed it when you look back…We were an energetic bunch in many ways and mother was so busy with the cooking and the cleaning and the housework until we grew up a bit and all got our jobs which must have helped.

The other was the back yard vegetable garden (her father’s):

The significant thing I think about the back yard was the vegetable garden because my father was a great gardener all his life and he really provided for our food requirements practically every year from the garden. And it was almost sacred ground. We weren’t allowed on it during the growing season.

The conservative features of family life persisted through Jean’s teenage years. The girls received no information “about the physical side of sex” and no encouragement to have boyfriends or bring them home. In contrast,

Gradually the boys brought their fiancées home, but it was always at that stage and then it became a very welcoming thing, and we practically always remember the family tea on a Sunday night that nearly always included the girlfriends of the boys.

Paternal authority and discipline came mostly in psychological form.

Well, he was a disciplinarian, and it was that that made it tough, not that we the girls were ever strapped. And I can’t ever remember dad getting angry with us in that way, but he had another way of getting angry and it was tougher than being thrashed I think or being strapped or smacked. We got smacked yes …. I can’t remember mother ever smacking us, but dad did. And there was a strap that hung beside the fireplace in the kitchen…. And he would just go silent. That was his way. And mother must have had that.

But as Jean conceded, everyone in the family “just accepted it, that that was the way, and that dad was the head of the household and that he mustn’t be balked in any way”.

Fourth, as in much of Otago and Southland at the time, when Jean was not in school or at home, she was involved in the local Presbyterian congregation: Sunday school, church, Manse social activities, and bible class camps. Jean was from a Presbyterian family – her father remembered going around the Taieri, where her grandfather used to preach, in a horse and gig. Her earliest memory was from before she went to school, the family tradition of trooping next-door on a Sunday afternoon:

…to hear the radio on a Sunday afternoon and it was a hymn session, hymn sing song I think, and we used to line up each Sunday afternoon and go in there and spend an hour with the people next door. And they became very interested in us and were very generous but the thing I guess I remember was the passing round of sweets from the sweet jar as part of my early memory.

Although she could not remember her mother or father ever accompanying the children, Sunday school and church were a major influence throughout Jean’s childhood and young adulthood to the extent that she felt “deeply involved spiritually, socially and physically”.

It became a real social side of my life, and I steamed ahead in lots of the social initiatives that were taken at that time and I would say that in the 1930s and into the early 1940s it dominated my life because we set up also a sports club at the church, a tennis club and we had regular social gatherings...

In my teen years it was First Church Invercargill and the Minister there was a man by the name of James Thompson, and he used to encourage the social activity of the young people and every Sunday night we would go to the Manse and sing around the piano there. Great, you know at times it had sixty young people squeezed into that room and we would sing our little hearts out … hymns that we got from the hymnal, and everybody would have a favourite and off we’d go and then I’d get a supper about ten o’clock and then we’d go home...

And I also got into the organisation of the bible class movement and gradually came through to President of the Southland Bible Class Movement there and I used to run bible class camps at Easter time for anything up to three or four hundred youngsters and do all the organisation for that and get the right people in to be speakers and that again would be the late thirties and early forties.

Finally, in terms of the lasting influences on her of this ‘direct and indirect enforcement of learning’, when asked whether she was like her mother in any ways, Jean replied, with the benefit of many years’ hindsight:

I’d like to think so, but I know that I’m very like my father…. I think the good things about my mother were her interests in each of us as children and trying to do the best for us I think, and I feel that that side of myself has led me into the whole counselling field early on; and my father’s side has given me the administrative skills or the start in the administrative skills that has led on in other ways.

Jean’s reflection on her psychological formation is again characteristic of an ‘obedience-subject’ living and learning in a ‘disciplinary society’: that gnawing feeling of never being good enough and, in response, the compulsion that one must constantly strive throughout life to prove oneself.

Well one of the things that I think I’ve inherited as a result of my, and it’s not just mine, I think most of the family have inherited, is a lack of confidence. And it seems strange to say that even when you look at the things that each of us have achieved but, basically, there is inside us a feeling that we’re not good enough. And I guess there’s been a striving there to say, you know, yes, we are good enough and I think that’s been part of our inheritance as well.

## Achievement Society and Childhood

The overall characteristic of children’s learning is that, in line with their development, they are absorbed in capturing the world by which they see themselves surrounded and of which they are a part. (Illeris, 2017, p. 8)

According to the Danish learning theorist, Knud Illeris, children expect to be guided by parents and other adults “as to what and how they should learn” (p. 189). This expectation continues through the adult-determined frameworks they experience in early learning and early school settings. However, what Illeris calls the ‘cultural liberation’ of late modern society has materially affected learning in childhood and youth. He identifies the disintegration or weakening of some norms and traditions, the pace of technological innovation and adoption, and the ways in which mass media and social media open children to:

…or more often almost force on them – a mass of impulses, including things such as catastrophes, violence and sex; experiences to which they have not previously had access, and which can have strong emotional influences on them, as well as introducing these things in advance of the formation of personal experience, making it more complicated for them later to acquire their own experiences in these spheres. (p. 189)

By the time children are in the youth phase of their life course, for most the identity process “is far more immediately important and far more urgent than academic learning” (p. 192). At the same time, though, “the demands on the formation of identity have undergone an explosive growth in line with cultural liberation” (p. 191) and social fragmentation. Because of this “young people must find their own way through their own choices” (p. 191) and struggle with a rapidly changing social world. In this social world, says Illeris, young people are faced with countless possibilities and choices, but also countless limitations given that only a tiny fraction of young people are likely to be able to pursue the idealised consumption-based lifestyles and life pathways they are bombarded with through the media. Yet, if we adults are committed to working in education with a non-romanticised view of childhood today, we surely need to ensure that education enables children to rehearse and embody the capabilities and capacities to navigate society as it is and as it is becoming.

In contrast with the excess negativity of the disciplinary society, Han argues that our present achievement society suffers from an ‘excess of positivity’ (p. 11). Earlier norms and traditions of negative self-discipline and self-constraint have not completely disappeared but now, in addition, the achievement-subject feels a compulsive freedom to achieve, to sample all available experiences. The achievement-subject feels compelled also to excessive work and performance but is, in Han’s phrase, ‘no-longer-able-to-be-able’ and suffers from solitary tiredness, creative fatigue, depression and burnout. Moreover, “excess positivity also expresses itself as an excess of stimuli, information and impulses” (p.12). Excess in turn affects cognition and attention, leads to continuous multitasking and an erosion of the unique human capacity for ‘contemplative attention’. Instead, the achievement subject experiences hyperattention; “the gaze errs restlessly and finds expression for nothing” (p. 15).

Han describes the *psychological* effects of the achievement society in broadly similar terms to the German sociologist Hartmut Rosa in his major book, *Social Acceleration* (Rosa, 2013). Rosa, however, identifies three *structural* dimensions of social acceleration. First, *technical acceleration*, “the intentional acceleration of goal-directed processes” such as transportation, communication, and production (p. 301). Second, the *acceleration of social change* by which he means the progressive shrinkage of the intervals of time in which one can anticipate stability of experience and expectation with respect to “fashions, lifestyles, work, family structures, political and religious ties, etc.” (p. 301). Third, the *acceleration of the pace of life* “represents a reaction to the scarcity of (uncommitted) time resources” (p. 301). Reactions may be in the form of stress and a lack of time; or an increase in the number of actions or experiences in each unit of time.

One of the pathologies of social acceleration is the constant fear of “getting left behind” (p. 316). Love, friendship, and achievement all need constant refreshment, renewal and improvement leading to restlessness and a “restructuring of the order of values as a result of problems with time” (p. 317). These include a tendency to focus on ‘putting out fires’ and short-termism which together “produce the widespread feeling that one no longer has any time for the ‘genuinely important things’ in life” (p. 317).

Now, while neither of these authors speaks directly of children and childhood, they are in my view accurately portraying key elements of the contemporary socio-economic and cultural milieu into which our children and young people are born, develop and grow according to Knud Illeris’s analysis. Equally, we know all too well from several local childhood monitoring studies (e.g., *Growing up in New Zealand* and *Youth 2000*) and system level public health data that children and young people in Aotearoa New Zealand, adolescents in particular, face multiple challenges to their health, wellbeing, belonging and identity. These are of orders of magnitude and complexity quite unimaginable in the childhood world of the early 20th century.

As Illeris summarises it, then, at the same time youth has become idealised and commercialised, “the personal and societal problems that attach to youth seem to be steadily increasing” (p. 191). While the trends are unquestionable, I think we can still find reason to be both optimistic and hopeful. But this does require us to develop a shared, plain language, non-romanticised and pragmatic educational ‘imaginary’ of how we want our children and grandchildren to be able to stand confidently, happily and healthily in their chosen adult worlds.

In a 2016 Deans’ Series lecture at the University of Melbourne, Linda Tuhiwai Smith reflected on the Māori experience of navigating thirty years of structural adjustment in education infused with a rhetoric of ‘choice’ (https://www.youtube.com/watch?v=7MaFf4Bgufk). She talked about: (i) Māori as an indigenous people negotiating consent to be educated as Māori and as a citizen of New Zealand without losing either their language or their culture; (ii) a non-romanticised Māori imaginary that seeks engagement with modernity and the settler state, as both self-determining sovereign entities and individual subjects in New Zealand; and, (iii) in the words of Mason Durie’s goals for Māori development, aiming to live as Māori, to be citizens of the world, and to be well. Smith incorporates these interwoven images of educational possibility in her phrase, ‘the twenty-first-century Māori subject’.

I believe that Smith is intentionally using the concept of imaginary here in much the same way as the Canadian hermeneutic philosopher, Taylor (2004). He distinguishes between social theory and social imaginary and uses the latter term:

(i) because my focus is on the way ordinary people “imagine” their social surroundings, and this is often not expressed in theoretical terms, but is carried in images, stories and legends. It is also the case that (ii) theory is often the possession of a small minority, whereas what is interesting in the social imaginary is that it is shared by large groups of people, if not the whole society. Which leads to a third difference: (iii) the social imaginary is that common understanding that makes possible common practices and a widely shared sense of legitimacy. (p. 23)

For Taylor, the imaginary “extends beyond the immediate background understanding that make sense of our particular practices” (p. 25) and gives us “a wider grasp of our whole predicament: how we stand to each other, how we got to where we are, how we relate to other groups and so on” (p. 25).

Smith also provides ‘a wider grasp’ of the Māori ‘predicament’ when she conceptualises an indigenous research agenda as a voyager chart (Smith, 1999, p. 117). She does so through the metaphor of Pacific ocean tides and the sea as a giver of life. The tides represent “movement, change, process, life, inward and outward flows of ideas, reflections and actions” (p. 116). Their four directions represent decolonization, healing, transformation and mobilization. The major tides are survival, recovery, development and self-determination. It seems to me that this is precisely what coalesces research, policy and practice in kaupapa Māori (and also ‘Pacific Way’) approaches to education as a whole. On my reading, and flipping momentarily to the metaphor of our conference theme as an ocean voyager chart, it is also precisely what is missing from our English medium aspirational research, policy and practice ‘tri-hull’, one that all too often gets blown off course by: (i) the shifting winds and tides of political ideology that seek short-term electoral advantage; and (ii) the habitual bottom trawling by the Pākehā 24-hour commercial news cycle and talkback radio for education clickbait, and the combined destructive effects of both of these on the quality of our public sphere discourse about children’s learning.

A perfectly reasonable basic expectation of state education is that the experiences it affords should enable children to develop sufficient autonomy to be able to navigate their early adult life choices and pathways through home, work and community. Such an expectation implies an appreciation by education research, policy and practice actors of: (i) how home, work and community function as social institutions; (ii) the knowledge, skills and dispositions that children need to acquire through early learning, schooling and post-compulsory education in order to be capable of exercising individual and collective agency in pursuit of their best life; and (iii) the past, present and foreseeable contextual factors most likely to disrupt those learning processes. That in turn requires us as a society to have an optimistic and hopeful educational imaginary at the centre of which stands a self-assured, culturally grounded 21st century Aotearoa New Zealand subject. Both hope and optimism are necessary if we are to meaningfully recognise children’s learning and appreciate that formal education is merely a subset of learning, not the other way around.

## Research, Policy and Practice

And so, to the theme of this year’s conference - the nature of the triangular relationships between educational research, policy and practice. In Jean Herbison’s childhood world, the triangle may have represented simply a plane geometric figure or the holy Trinity. In today’s culturally pluralist world, children are just as likely to encounter the triangle as an example of technological innovation in Tāniko weaving border design, or as a personalised narrative of Polynesian tattoo art in the form of a niho, the shark’s tooth.

This alerts us to the need as adult educators to critically examine the untested assumptions we may be bringing to a beguilingly simple and inviting image that conveys the impression of a strong, enduring, relational unity of purpose among three clearly defined institutional actors. But even at the most basic level, this is a partial and distorted depiction of those involved. Where, for example do professional associations, iwi, hapū, rūnanga, Urban Māori Authorities, businesses, think tanks, lobbyists, charities, gaming trusts, voluntary organisations, faith communities, and social enterprises fit in our triangle given that most or all are active in one or more of education research, policy and practice (e.g. Ball & Junemann 2012) understood as discursive and recursive processes? Most importantly, where do children and whānau fit, with what quality of recognition, and with what authority and influence of individual and collective voice?

In actuality, these institutional groupings are often only loosely coupled clusters of related activity. The particular actors in them need to re-establish common understandings and mutual trust at each significant new encounter, while also recognising that the communities in which their joint educational reform is planned, especially socio-economically disadvantaged and culturally minoritized communities, may have long experience of relational, structural or contextual distrust in the heady promises of reformers (Schultz, 2019). And, as Flyvbjerg has argued in the context of urban local authority transport planning, making sense of such dynamics requires a realpolitik approach to “understanding rationality and politics, and with the power-as-strategies-and-tactics view of power, the dynamics of conflict and struggle become the center of analysis” (Flyvbjerg, 1998, p. 6).

**Fig. 1:**
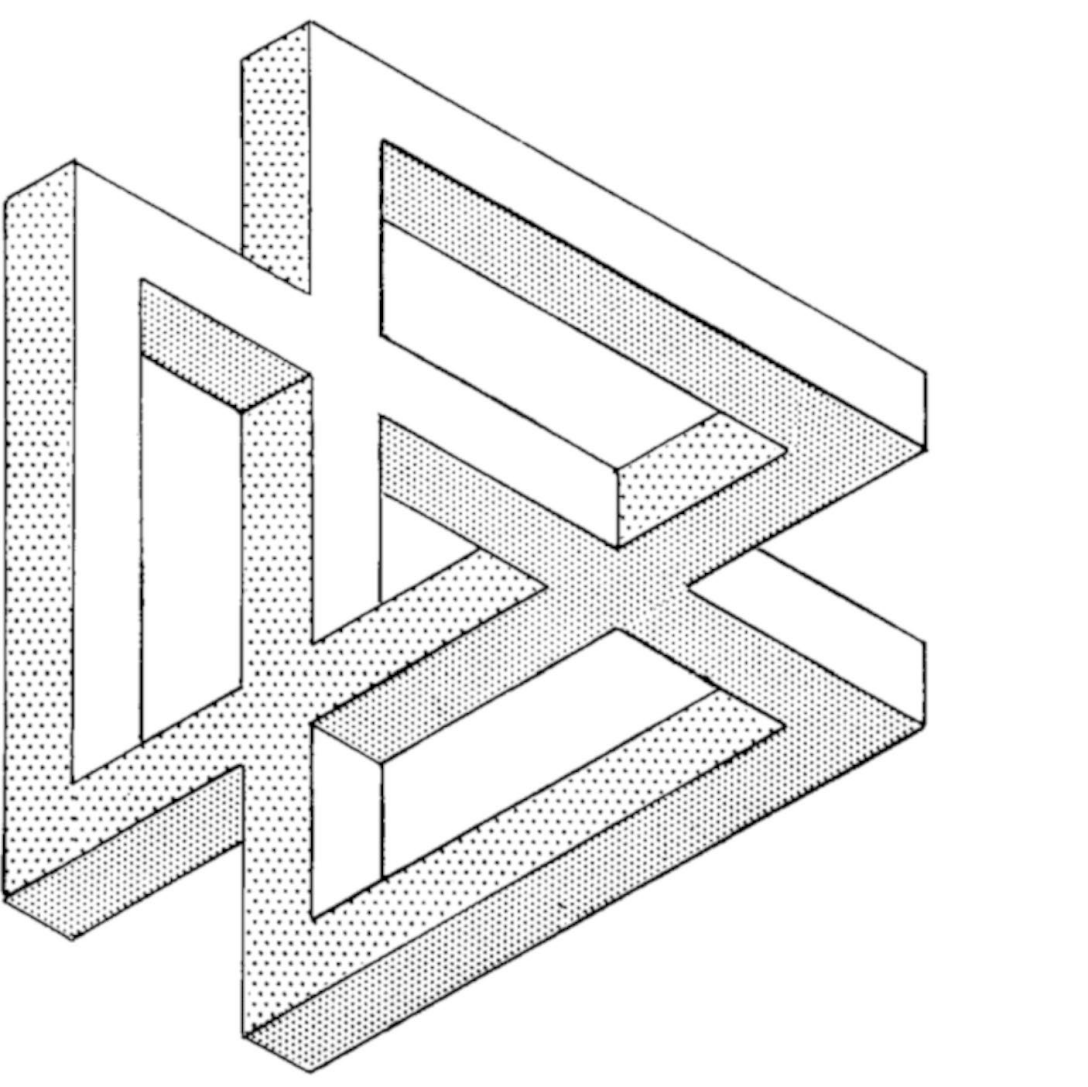
Diagram of structure with multiple impossibilities (Penrose & Penrose, 1958, p. 32)

This suggests to me that an appropriate image of the practical relationships between education research, policy and practice is not that of a two-dimensional closed figure, but rather a three-dimensional impossible triangle (Penrose & Penrose, 1958). Indeed, given the number and diversity of institutional and community actors in our space today, it might be more accurate to extend the metaphor to one of a structure with multiple impossibilities (Fig. 1).

Such an image, I suggest, gives us a far more realistic sense of what occurs when we ask research, policy and practice to combine. It encourages us to be both critical and pragmatic in debating our ways forward. The history of educational research and reform, here and overseas, tells us much the same. For example, the American educational theorist, Elliot Eisner, argues that the propositional language favoured in many education policy texts and research reports struggles to capture or resonate with the day to day realities and concerns of practitioners (professional and whānau).

Because the realities of the classroom and of social life in general are, at base, an array of qualities for which meanings are construed, they will always present more to the perceptive teacher than propositional language can ever capture. The particularity of a set of conditions, the uniqueness of an individual child, the emotional tone of something said in love or in anger, the sense of engagement when a class is attentive will always elude the language of propositions. Yet it is precisely these qualities that the teacher must address in his or her own work. The language of propositions is a gross indicator of such qualities: it cannot capture nuance – and in teaching as in human relationships, nuance is everything. (Eisner, 2005, p. 93)

Equally, the American curriculum historian Herbert Kliebard observes that a rhetoric and spirit of reform, however well intentioned, all too often founder on the rocks of teachers’ ‘practicality ethic’:

Educational reforms involving changes in teaching practice fail with such monotonous regularity because enlightened reform rhetoric and the generosity of spirit that impels people to change things for the better simply comes into direct conflict with institutional realities. Good intentions and even competence notwithstanding, teachers are absolutely required to maintain a precarious order, and only the very courageous are willing to risk its loss. (Kliebard, 2002, p. 137)

More directly relevant to today’s focus on children’s learning, perhaps, a gloriously utopian local illustration of exactly Kliebard’s point is to be found in the 1970s (the high point of our Pākehā social democratic progressive schooling sentiment). Here, an ‘enlightened rhetoric and generosity of spirit’ informed the new architectural design and construction brief for the whānau house secondary school, with its explicit design for learning:

The processes of learning have always extended beyond a formal distinction of pupil, teacher and classroom. Learning is understanding and interpreting one’s social and physical environment so as to recognise and work to maximum advantage with one’s own talents, capacities and intelligence. A school must encompass this extended concept of learning. (Department of Education, & Ministry of Works & Development, 1975, p. 8)

These schools were to be planned be “guidance-centred rather than subject oriented”, to be “a stimulating, challenging and satisfying place to live in and work in all day”, to encourage “purpose, spontaneity and a feeling of belonging” and to be “the antithesis of boredom, regimentation and alienation” (p. 7).

The aims are two-fold: to give each pupil insights, knowledge and experience so that he [sic] can understand himself and the people he lives and works with, in both the smaller and larger community; and to make the most of what he can do personally that is unique and vital to him. Dignity and self-esteem are essential to every human being, as is the feeling of belonging. Within the extended family of each whanau house, pupils will feel accepted and of value…. The whanau house is designed to be a place where pupils and teachers want to be and where the community is made welcome. It is a logical framework for preparing young people for life in our society. (p. 7)

However, as Rae Munro concluded following a two-year case study of efforts to establish a single whānau unit at Penrose High School, unless consideration of institutional realities (and their socio-historical contextualities) is integral to the initiative, success is unlikely. In this instance, he identified the school’s established expectations of teachers and students: “A degree of autonomy had been assumed which could not be realised in practice. Without such necessary independence the Whanau staff had been presented with the impossible task of literally restructuring secondary schooling” (Munro, 1980, n.p.).

The challenges of achieving the distance necessary for genuinely critical reflection and critically informed action concerning what will benefit children and their learning most, are only compounded by the reality that some of our institutional actors at system level are constitutionally required to be politically deferential, or politically neutral, or politically blind, while others make strategic and tactical choices about whether even to participate and on what terms. The former Chief Science Advisor identified the Ministry of Education as one government department that was particularly prone to conflating facts and values as evidence in policy formation (Gluckman, 2013), as he put it. Irrespective of whether we believe in the possibility of separating facts and values in education, I don’t think it is too harsh to suggest that the Chief Science Advisor could reasonably have levelled much the same accusation at parts of the education research community, the education profession, and at fractions of civil society.

Yes, to be sure, we now have a Chief *Education* Science Advisor, but it stretches credibility to suggest that this one part-time, goodwill reliant position is adequate to address the magnitude of structural and political challenges involved. And in any event, if we accept the proposition that underpins the newer social science sub-disciplines such as childhood studies, sociology of childhood and children’s geographies, namely that ‘children are experts in their own lives’, then we need to significantly increase the extent to which their voices, experiences and insights are heard, and acted on, in setting our educational research, policy and practice agenda.

Children and young people identified and shared areas of their education experience that could be improved. We heard common themes about marginalisation and discrimination from: tamariki and rangatahi Māori; children and young people who are Pacific Peoples; those with disabilities; and those who have been excluded from school. A diverse range of children and young people told us their unique learning needs are often not being met. Some are aware of the potential contribution that school can make to their lives, but feel that potential is withheld or undermined by factors such as bullying, uncertainty, attitudes of teachers, issues at home, and experiences of racism or lack of cultural understanding. We heard these stories particularly from children and young people in alternative education settings, but also from children and young people in mainstream schools and kura kaupapa Māori. (NZSTA, & OCC, 2018, p. 43)

## Forms of Life

At this point then, it may be helpful to move from the concept of educational research, policy and practice as the idealised alignment of disparate institutional activity systems, and toward the German philosopher Rahel Jaeggi’s concept of forms of life (Jaeggi, 2018).

Jaeggi defines forms of life as clusters of related activities. For her, they concern the “cultural and social reproduction of human life” (p. 3).

Forms of life are complex bundles (or ensembles) of social practices geared to solving problems that for their part are historically contextualised and normatively constituted. (p. 29)

In terms of the research, policy and practice triangle as a problem-solving form of life, what interests us is the rationality of the dynamics of its development. The question of the extent to which it is successful concerns not so much its content, but rather its rationality and success as “an ethical and social learning process” (p. 29). The focus of critique of forms of life is not simply convictions and beliefs (in our case about education) but the substantive “conditions of life that human beings can shape and transform” (p. 29).

For this reason, Jaeggi argues forcefully against ‘ethical abstinence’, the stance that we should just accept the validity of different modes of research, policy and practice as existing alongside each other. Instead, she argues, we should be evaluating them in terms of: (i) their ability to solve the problems or periodic crises that these forms of life identify — in our case, do they help children to flourish in and through their learning?; and also (ii) the ideologies and historically contextualised assumptions that frame these problems and crises in the ways they do — topically, for example, how does non-attendance at school come to be framed as an issue of youth, parent and professional pathologies, and not a deeper analysis of the ways in which contemporary schooling’s obsession with outcomes, standards and benchmarks contributes to the reification of students and the alienation of children from their learning?

Jaeggi distinguishes three approaches to critique of forms of life: (i) internal; (ii) external; and (iii) immanent critique. Internal critique would take the framing of educational research, policy and practice problems and their solutions as given (e.g., our now decades long pattern of small budget, principal-agent contract evaluations of new policy initiatives, and Education Review Office national evaluation reports). External critique would apply external standards to internally framed problems and solutions (e.g., our cyclical Pisa, TiMMS, PiRLS envy/anxiety rituals and associated policy borrowing from supposedly better performing OECD countries overseas). In contrast, Jaeggi argues that immanent or transformational critique is “an ethical learning process” (p. 31). Moreover, “the evaluation of forms of life should find its criterion in the subject matter of the problem or in the success of problem-solving processes” (p. 31). And here, I think, the paradigm case for us in education is Kaupapa Māori led research, policy and practice, as evidenced in the recent Te Pae Roa report on the future of Kaupapa Māori and Māori medium education, which succinctly defined ‘the problem’ as follows.

Te Pae Roa have come to the view that the issues raised are largely symptomatic of a systemic issue – the Crown’s assumed ownership and governance over Kaupapa Māori education and the use of mātauranga Māori (inclusive of te reo Māori) in English-medium settings. (Te Pae Roa, 2022, p. 9)

## Reification, Alienation and Recognition

Today the discursive threads of English-medium educational research, policy and practice are colourfully adorned with sparkling costume jewellery gems such as ‘decolonisation’, ‘Te Tiriti-led’ ‘kawangatanga’, ‘language, culture and identity’, ‘wellbeing’, ‘te whare tapa whā’, ‘whānau’, ‘mokopuna’ and ‘ākonga’. It seems reasonable to ask whether and to what extent these represent yet more tactical Pākehā virtue-signalling or are, instead, the ‘green shoots’ of a critically aware, deepening commitment by Tangata Tiriti to transformation of the basis of learning to one in which children and childhood are given the holistic recognition they deserve.

Specifically, can educational research, policy and practice as a community manage to find ways to abandon the collective English-medium schooling, cognitive outcome-driven obsessions of the last four decades? In all my accumulated sadness and frustration, I cannot help but liken this obsession to constantly measuring, weighing and intensively rearing livestock for (the labour) market. Such an emotionally charged response may be understandable, but of course it serves no practical use. A more practically beneficial approach may be to look deeper and critically beyond the surface features of educational activity, to both their material effects on children, and the deeper causes of these. Here, I believe, the concepts of reification, alienation and recognition are of considerable practical value to us as educators.

Both Jaeggi and Axel Honneth view the central concern of modern social life as freedom through self-realisation; the ability and opportunity to live in a way that is appropriate to pursuing one’s best life. They also agree that: (i) we can only achieve our freedom socially with and alongside others; (ii) the norms we act on are immanent in our forms of life and social institutions; and (iii) these norms shape our social traditions and routine practices. In the case of our 2022 conference, that means the (often loosely-, occasionally tightly-) clustered activities of educational research, policy and practice as a form of life. For Honneth (Honneth, 2008), the pathologies that these social activities produce may lead to reification. In Jaeggi’s case (Jaeggi, 2014) to alienation.

## Reification

In his recasting of the concept of reification from its historic use in the processes of production and commodity exchange, to contemporary anthropological and institutional relations, Honneth describes it as a losing sight of, a forgetting, or denial of our (antecedent) recognition of the essential humanity of other persons (or groups of persons), and of the natural and social dimensions of the world that in turn are of value to those persons. For Honneth, the social sources of reification, the conditions that enable forgetting or denial of recognition, include practices where observation of ‘the other’ becomes an end in itself or is guided by convictions or ideologies that lead to a denial of recognition, such as stereotyping. Another source is more personal in its effects: that is, denial “that our desires, feelings, and intentions are worthy of articulation” (2008, p. 82). This in turn can lead to self-reification when we believe that our ‘psychic sensations’ are merely objects to be observed, produced, performed or portrayed publicly through institutional practices. Social practices and institutional arrangements can consequently promote both reifying, and self-reifying, behaviour.

## Alienation

Jaeggi (2014) describes alienation as a ‘relation of relationlessness’, or the absence of meaningful relationship to oneself and to others. Alienation can take the form of ‘living one’s life as an alien’ as she puts it, and as a ‘disturbed appropriation of self and world’. In her analysis of the theory of alienation, Jaeggi (2014) identifies three problematics, First, it shows how individual lives can ‘go wrong’. Apathy, indifference toward life and a feeling of powerlessness negatively affect the individual’s disposition toward the chances of achieving a good life, in general, and personal autonomy, in particular. Second, alienation impairs one’s ability to identify with a form of life, to realise oneself in it and to make one’s life one’s own. Third, it helps describe and explain the workings of capitalist society at large (to which we might add, following Honneth, the social institutions that comprise a particular capitalist society such as ours). Taken as a whole, Jaeggi’s reading of alienation is centrally about “individuals’ relations to the social relationships, practices and institutions within which they lead their lives” (p. 216). On her analysis, “if self-alienation is also alienation in and from the social world, then the problem … can only be solved in, not beyond the world of social practices” (p. 217). She concludes by making a link between the constitution of subjects, and the constitution of institutions, and asks:

How must institutions be constituted so that individuals living within them can understand themselves as the (co-authors) of those institutions and identify with them as agents? What would social institutions look like that could be understood as embodiments of freedom? (p. 220)

This is a challenging enough pair of questions if we view the institutions of research, policy and practice as formally separate, self-governing activity systems. How much more complex are they when we conceive of the clustered activities these institutions undertake as a form of life? Honneth addresses these kinds of recognitive social relations in his two, linked major works, *The Struggle for Recognition: The moral grammar of social conflicts*, and *Freedom’s Right: The social foundations of democratic life*.

How do persons develop and maintain their identity, their sense of themselves as practical moral beings with unique characteristics and distinctive places in the social world? The basic answer that Honneth proposes is: individuals only become who they are in and through relations of mutual recognition with others. In short, persons gain subjectivity only intersubjectively. Only when individuals receive positive acknowledgement of their own personal traits, standing and abilities can individuals begin to see themselves as others do and thereby gain an efficacious sense-of-self. (Zurn, 2015, p. 6)

## Recognition

Simply put, *Struggle for Recognition* (Honneth, 1995) is focused on the individual’s experience of striving and learning to live within society’s institutional structures, while *Freedom’s Right* (Honneth, 2014) is about the practical encouragement of social justice and the ways in which society’s institutions encourage or inhibit the individual’s self-realisation. In these terms, it strikes me that the moment we have reached in education research, policy and practice today is one of clarifying what we mean by mutual recognition and institutional self-realisation. However, the possibilities that we all hope for will only provide lasting benefits for children and their learning if we are prepared to do the intellectually and emotionally difficult work of: (i) socially critical evaluation of our current approaches to defining and addressing educational problems or crises; *and* (ii) taking practical action to reframe these more productively towards the development of socially just educational arrangements for learning.

Honneth identifies the patterns of intersubjective recognition as love (primary relationships), rights (legal relations) and solidarity (community of value) (Honneth, 1995, Fig. 2, p. 129).^2^ These recognition patterns cover developmental growth from childhood to adulthood, and in settings from the intimacy and privacy of home to the public sociality of workplace and civic sphere. Primary loving relationships address needs and emotions, provide emotional support and lead to basic self-confidence. Legal rights encourage moral responsibility and provide for self-respect. Solidarity with others in one’s various social groupings and communities encourages the development of traits and abilities and with these, self-esteem. Disrespect in the form of physical abuse threatens one’s physical integrity, denial of rights or exclusion one’s social integrity, and denigration or insult one’s honour and dignity. I don’t think it takes much effort to map these abstract patterns of relations of recognition onto our collective knowledge of what education in Aotearoa New Zealand does well, and not well at all, for children and their learning. But if these are the material possibilities for recognition, misrecognition, and disrespect that children must learn to navigate, what are the institutional arrangements that may provide better support for individual self-realisation, freedom and enhanced possibilities of leading an ethical life?

On this issue, Honneth argues that “the ‘official’ spheres of law and morality merely serve as a means of detachment or reflexive examination” (Honneth, 2014, p. 127). By this, I think he means that the sorts of statements we rely on in terms of providing statutory guidance for adults are of limited utility and effect in terms of what actually takes place in learning interactions involving children. These statutory instruments include: (i) children’s rights generally (e.g., the United Nations Convention on the Rights of the Child, the United Nations Declaration on the Rights of Indigenous Peoples, the United Nations Convention on the Rights of Persons with Disabilities); (ii) their rights to an education in Aotearoa (e.g., The Education and Training Act 2020); and (iii) the derived obligations of early learning service and schools to operationalise these, and report publicly on progress in fulfilling them.

For Honneth, we can only guarantee freedom (i.e., the possibility of self-realisation and an ethical life) by working towards “the spheres of action in which mutually complementary role obligations ensure that individuals can recognize each other’s free activities as conditions for the realization of their own aims” (p. 127). Now, if all this appears hopelessly abstract and removed from the pragmatic concerns of: (i) systemic education research, policy and practice; and (ii) everyday interactions among learners, educators and whānau, let me progress towards an end point, or better for our purposes today, perhaps, an intermission, by identifying some examples from our education history, where I think critically aware and critically engaged educators in Aotearoa have managed to establish precisely the sorts of complementary role obligations that provide for mutuality of recognition and greater freedom in children’s learning.

## ‘Walking Backwards into the Future’

Educational studies, including applied critical theoretical studies, have been in relative decline in English-medium university education faculties in New Zealand since at least the early 2000s. Their continuing relevance to practical spheres of action at the nexus of education research, policy and practice in today’s cognitively weighted and assessment outcome dominated educational imaginary is commonly seen to be at best outworn, at worst too awkwardly questioning by far. But there is one notable exception, and we would do well to consider why this might be the case.

Smith (1999, pp. 185–189) discusses some of the commonalities, differences, and tensions between Kaupapa Māori research and Western academic critical theory. She argues that through: (i) localisation of the emancipatory goals of critical theory; (ii) strategic positioning of the researchers within these local struggles; (iii) personal positioning and identification of researchers as Māori; and (iv) involving whānau communities in the orientation, decision-making and work, Kaupapa Māori research has managed to be critical, emancipatory and relevant in the sense of facilitating practical, community-oriented responses to the problems it surfaces.

This begs the question whether locally focused, contextualised and reflexive critical theoretical efforts directed at children’s learning in English-medium settings can contribute tangibly to what we would all want to see as the more closely interwoven discursive threads of education research, policy and practice, for the benefit of all children: tangata whenua, tangata Tiriti and tauiwi. I would argue fervently that it is, and on much the same ethical basis to that identified by Māori researchers undertaking Kaupapa Māori research. For English-medium contexts then, we might usefully and humbly reappropriate that indigenous ethic of relationship as follows.

Any educational research that involves communities should set out to make a positive short or long-term benefit for the people involved through working respectfully with communities, sharing knowledge and processes.

Fortunately, we do not start here with a blank slate. Some English-medium educational research, policy and practice initiatives that have made the most material difference to the learning of children and young people over the decades, have been those where their proponents, on behalf of children, have adopted a broadly critical and reflexive stance toward the prevailing education settlement of the time and have taken more purposive and, often, courageously different paths instead. Let me offer here a few examples of: (i) learning site-based case studies; (ii) national policy innovation; (iii) cultural responsiveness; and (iv) opportunities for children’s voices on learning and education to be heard.

### Case Studies

Case studies are an essential part of our shared education learning repertoire because, as Flyvbjerg puts it, “A discipline without a large number of thoroughly executed case studies is a discipline without systematic production of exemplars, and a discipline without exemplars is an ineffective one” (Flyvbjerg, 2001, p. 87).

Elwyn Richardson explains in the Introduction to *In the Early World*, that his richly illustrated presentations of children’s learning (which give equal weight to their learning artefacts and his pedagogical narrative) are integral to his “attempts to understand children, especially their desire to express themselves in their own natural ways” (Richardson, 1964, p. xiii). The adult narrative documents how he and the couple of other teachers at Oruaiti school learned from the children, while the copious photographs, prints, ceramics, and creative texts that make up the bulk of each chapter exemplify how the children learned to express their individual ‘idioms’ through art, music, movement, drama and language, with active support for Richardson’s progressive methods from the children’s parents. As Richardson said of the children: “They were my teachers as I was theirs, and the basis of our relationship was sincerity” (p. xiii).

The equally but differently rich, multimedia classroom research projects undertaken collaboratively by Graham Nuthall and Adrienne Alton-Lee and summarised in *The Hidden Lives of Learners* (Nuthall, 2007), revealed much about how children navigate the curriculum in use and about their multiple relations of mutual recognition and misrecognition in the classroom. The studies produced insights about children’s learning that were likely only possible because of Nuthall’s decision, prompted by Alton-Lee’s PhD research, to shift the focus of his own research from the teacher to the student. This is a ‘case study’, then, not just of children’s learning itself but also in the transformative sense of researchers (to which we would want to add policy and practice professionals) gradually coming to a meticulously observed and overheard appreciation that children’s unromanticised lived experiences within routine classroom relations must form the evidence base of better decisions about how to support their learning.

*Colouring in the White Spaces* is a reflexive account of Ann Milne’s professional journey over several decades as an urban school principal to answer the question about whether schools can create the conditions, and remove the barriers, so that involuntarily minoritized students may: (i) live their culture while at a school that operates within a majority culture system; (ii) develop strength in their cultural identity; and (iii) learn through culturally-specific ways of knowing (Milne, 2016). Milne’s account shows how, with culturally grounded love, care and high expectations, and working with the agreement of whānau communities, it is possible to empower children to overcome the deep psychological trauma (i.e., reification, alienation and misrecognition) of being a minoritized child in a racist English-medium schooling system. She concludes:

White spaces can be colored in by practice that gives a community voice, that listens, that responds, and that is underpinned by the cultural knowledge and beliefs of its people. This practice conscientizes whānau to resist the status quo, to demand more and to transform the educational experiences of our children. (p. 205)

### National Policy Innovation

It is perhaps difficult to recall in 2022 just how inflammatory to many educators were the conceptual framing, calculus and lexicon of The Treasury’s 1987 post- election briefing to government on education ‘issues’.

Parents will balance the judgments of the benefits and costs of the education to their children from various sources of ECS [Early Childhood Services] against the benefit and costs to them of the custodial function. There may be difficult trade-offs — for example, a higher standard of living against less parent/child contact time. Hence, in a hypothetical free-choice situation where the parents are fully informed of the pertinent benefits and costs to all parties of all options, the parents will not necessarily optimise the net benefit to the child. The trade-off between benefit to the child and benefit to the parents is natural and inevitable. However, some parents will ‘fail’ as parents, not in that they make a trade-off, but in that they unduly weight their own interests against those of the child. This may not always be deliberate. It may be because the long-term effects of different forms and sources of education and care are not realised and assessed. (The Treasury, 1987, p. 54)

Given the lasting impact of the neoliberal economic dogma of informed market choice on all education sectors, policies and policy actors over the last thirty-five years, the enduring educational values and children’s learning-centred legacies of the contracts negotiated with the Ministry of Education that established the Early Childhood Curriculum Project (i.e., *Te Whāriki* ) and the *National Education Monitoring Project* (NEMP) are all the more remarkable.

These stories have been told by previous Herbison lecturers but what I want to emphasise here today is simply that in their different ways, both initiatives have placed children and their learning at the heart of the work. Both have refused to remain ‘ethically abstinent’ in the face of hegemonic system pressures to conform. Both have endeavoured to persuade other policy actors (whānau, community, professional and polity) of the imperative of viewing children and their learning in the round. Through several generations of guardianship, both initiatives have survived, more or less intact, periodic New Public Management polity reviews, and the bystander carpings of both market-liberal and neo-conservative echo chambers in society at large.

Thankfully (at the time of speaking) both remain steadfastly committed to not doing anything that would end up disrespecting, misrecognising, forgetting, denying, or inhibiting children’s entitlement to develop self-confidence, self-respect and self-esteem through rich curriculum and rich assessment practices, and the equally rich pedagogies these require to engage and absorb children in their learning in formal education settings.

### Cultural Responsiveness

From its inception, the Early Curriculum Development Project that resulted in the bicultural framing of the first national early childhood curriculum, *Te Whāriki*, consciously recognised an abundance of philosophies about childhood, children and their development in relationship with whānau and educators. This, I think, in great part explains its consolidation as an enduring ‘learning imaginary’ for the sector.

The problems of cultural non/misrecognition addressed through *Te Kotahitanga* in secondary schooling (and related polity- and community-led initiatives since), and *Developing Mathematical Inquiry Communities* in primary schooling, have been qualitatively different: disrespect, denial of rights, exclusion, denigration and insult. Their proponents (led by Russell Bishop and colleagues, and Bobbie Hunter and colleagues respectively) have worked tirelessly, over decades now, to challenge orthodox, deeply sedimented English-medium learning imaginaries (and the curricular, pedagogical and assessment repertoires that sustain these) in schools with large numbers of involuntarily minoritized learners. In doing so, they have been required to navigate often-sceptical research, policy and practice hegemonies, to the point where the ‘scaled-up’ evidence of the transformative effects of ‘normalising’ culturally responsive pedagogies and culturally embedded funds of knowledge, is overwhelming.

Equally, I think, in these projects there has been a remarkable generosity of educational spirit by Māori and Pasefika towards their Pākehā colleagues as they support us to expand our repertoires (knowledge, skills and dispositions) for facilitating and supporting children’s learning. This generosity of spirit alone should keep those of us in the Pākehā majority alert to the potential dangers of us enacting forms of recognition of children and their learning that maintain rather than disrupt unequal relations of power and domination. Drawing directly on McBride’s discussion of the politics of recognition and efforts to overcome the ‘recognition deficit’ (McBride, 2013, pp. 35–40), for example, we need to appreciate that: (i) children desire to be recognised in both universal and particular ways, as members of certain groups, certainly, but also for how they see themselves as individuals; and these desires may at times be in conflict; (ii) our aspirations as educators to become more culturally sensitive and responsive to children’s learning may lead us to view everything through a lens of ‘the exotic’ and this too can be oppressive and disrespectful; and (iii) we in the majority abjectly fail to see the true nature and extent of the structural problems of English-medium education if we believe that us ‘granting’ recognition of children’s culture is the answer, because this leaves our power to grant or withhold recognition intact and unchallenged.

### Providing Opportunity For Children’s Voices to be Heard

Over the last three decades, education research, policy and practice have begun to address the rights of children more explicitly and intentionally. This is in no small part because Aotearoa New Zealand is a signatory to major rights accords such as the *United Nations Convention on the Rights of the Child* (UNCRC), the *Convention on the Rights of Persons with Disabilities* (UNCRPD) and the *Declaration on the Rights of Indigenous Peoples* (UNDRIP) and must report to the UN periodically on progress in their implementation through statute, policy and institutional practice. Increased attention is now given to those articles in UNCRC that specify children’s rights to express their views on matters of interest to them and to have those views acted on by adults. Broadly progressive developments along these lines are reflected in, for example: (i) the establishment of the Youth Advisory Group to the Minister of Education, (ii) a recommendation from the Taskforce to Review Tomorrow’s Schools that the Children’s Commissioner should review the adequacy of children’s representation in school governance, and (iii) a burgeoning of so-called ‘student voice’ research.

There are encouraging recent examples of enabling diverse children’s authentic voice and participation in educational research using a variety of approaches: notably *Education Matters to Me* undertaken by the New Zealand School Trustees Association and Office of the Children’s Commissioner (2018), *Children’s Informal and Everyday Learning at Home During COVID-19 Lockdown* undertaken by Massey University and the New Zealand Council for Educational Research (Bourke et al., 2021), and *Conceptualising Māori and Pasifika Aspirations and Striving for Success (COMPASS)*, undertaken by NZCER and the University of Auckland’s Melinda Webber (Alansari et al., 2022).

Nevertheless, soliciting children’s views and providing avenues for their voices to be heard (in research, policy and/or practice) does not equate to recognition in the fullest sense of the concept, either for children as subjects or persons, or for their development and learning. For Honneth (1995), legal rights are a form of cognitive respect. Rights ascribe positive status to an individual as an equal member of a social community and encourage personal moral responsibility toward other members of the community in general. Just as importantly, “we can only come to understand ourselves as the bearer of rights when we know, in turn, what various normative obligations we must keep vis-à-vis others, [when] we recognize the other members of the community as the bearers of rights” (p. 108). This, I think, is a more complete and educationally valuable conception of the rights of the child, not least because it tightly couples together recognition rights and responsibilities.

And so, in a way, we are back again to Jaeggi’s two questions. Effectively, for our purposes, if we are to minimise the likelihood of alienating children from their learning: (i) How do we create the conditions so that children understand themselves as agentic co-authors of the educational institutions they attend? and (ii) If such institutions are to embody freedom for children, what would they necessarily look like?

Noddings (2003) famously argued that happiness in schools and classrooms should be a major aim of education.

One purpose of schooling should be to develop the intellect, but that does not mean to stuff the heads of children with material arbitrarily chosen by experts and designed to rank and sort them. It means rather to guide students toward the intelligent use of their intellectual capacities in both personal and public life. It means equipping them with the power to evaluate and direct change, to resist harmful changes and to promote those that contribute to human flourishing. Almost any subject matter of genuine interest to students, well taught, can contribute to this end. (Noddings, 2003, p. 260)

Hartmut Rosa has argued that the effects of social acceleration and alienation are mitigated when we are able to develop ‘resonant’ relationship with aspects of our natural, social and cultural worlds. Such resonance is formed through “affect, emotion, intrinsic interest and self-efficacy” (Rosa, 2019, p. 174). Resonance is a responsive relationship with the world through which we remain open to being affected by our world. In a similar vein, Iris Murdoch has written about the contribution of ‘the good’ to enable us to discern a new reality beyond surface appearances.

In intellectual disciplines, and in the enjoyment of art and nature we discover value in our ability to forget self, to be realistic, to perceive justly. We use our imagination not just to escape the world but to join it, and this exhilarates us because of the distance between our ordinary dulled consciousness and an apprehension of the real. (Murdoch, 1970, p. 90)

All the local initiatives just referred to are, in one way or another, concerned with enabling children to develop happiness, resonance, enjoyment and exhilaration in learning. Their originators have: (i) engaged determinedly in a critical yet practical realignment of research, policy and practice over time to address the structural educational shortcomings they have perceived; (ii) provided learners with positive, engaging and affirming ways to explore their natural, cultural and social worlds; and (iii) inspired educators in all parts of our education form of life to maintain the original, contextually nuanced spirit of reform across successive generations of children. In doing so, they have collectively given children a precious lived experience of what it is to enjoy freedom to learn.

Heoi anō.

He whakatauki mō tatou:

I orea te tuatara ka patu ki waho.

Nō reira, tēnā koutou, tēnā koutou, tēnā koutou katoa.

## Footnotes

1 Herbison, J. M. (1993), October 02 & 03. Interview by S. Fowke [Tape recording]. Dames Oral History Project (OHint-0046/19). Alexander Turnbull Library, Te Puna o Mātauranga o Aotearoa National Library of New Zealand, Wellington. (All extracts used with written permission of the Curator).

2 As Honneth’s (1995) translator, Joel Anderson, points out, to appreciate the arguments it is important to understand how Honneth uses certain terms in the original German language: to recognise individuals or groups is not merely to acknowledge their existence or presence but “to ascribe to them some positive status” (p. viii). In Honneth’s analysis this ascription is of love, respect and esteem. A failure to recognise is to ‘disrespect’, which can include “humiliation, degradation, insult, disenfranchisement, and even physical abuse” (p. viii). Interpretations of what is good or right in an ethical sense are based on the “substantive customs, mores, or ethos of a particular tradition or community” (p. viii) or its practices, while an ethical life refers to integrated social arrangements in which norms and values are embodied in “the basic attitudes and ways of life of members of the community” (p. ix).
